# Morphologic Mimickers of Seborrheic Keratoses: Cutaneous Lesions Masquerading as Seborrheic Keratoses

**DOI:** 10.7759/cureus.18559

**Published:** 2021-10-07

**Authors:** Darlene Diep, Antoanella Calame, Philip R Cohen

**Affiliations:** 1 Medicine, Burrell College of Osteopathic Medicine, Las Cruces, USA; 2 Dermatology/Dermatopathology, Compass Dermatopathology, San Diego, USA; 3 Dermatology, Scripps Memorial Hospital, La Jolla, USA; 4 Dermatology, University of California, Davis Medical Center, Sacramento, USA

**Keywords:** verruca, xanthoma, squamous, seborrheic, melanoma, keratosis, cell, carcinoma, basal, actinic

## Abstract

Seborrheic keratosis is an epithelial-derived benign neoplasm, which presents as a solitary tumor or multiple lesions. It is an acquired skin tumor that is frequently observed in older individuals. Benign neoplasms, as well as premalignant or malignant tumors, can mimic the clinical appearance of a seborrheic keratosis. A man presented with a chronic lesion on his abdomen that had changed in the color and size. The suspected diagnosis was a seborrheic keratosis. However, the microscopic evaluation of a biopsy tissue specimen established a diagnosis of a pigmented squamous cell carcinoma in situ. In addition to squamous cell carcinoma in situ and squamous cell carcinoma, other malignant tumors, premalignant lesions, and benign lesions can mimic a seborrheic keratosis. If a patient presents with a presumptive seborrheic keratosis that has changed in appearance and for which malignancy is also suspected, a biopsy may be helpful for diagnostic clarification to either confirm that the lesion is indeed a seborrheic keratosis or to establish the diagnosis of the lesion that mimics a seborrheic keratosis.

## Introduction

Seborrheic keratosis is a benign epithelial tumor. It typically appears as a flat or raised, often keratotic, plaque. It can present as a solitary lesion or multiple tumors [1]. Many skin lesions can mimic a seborrheic keratosis. These include not only benign tumors, but also malignant neoplasms. They also include precancerous skin lesions [1-19].

A man with a lesion on his abdomen that increased in size is described. Although the clinical impression was an irritated seborrheic keratosis, microscopic examination of the biopsied tissue specimen revealed a squamous cell carcinoma in situ. Other neoplasms whose appearance can mimic that of seborrheic keratosis are reviewed.

## Case presentation

**Figure 1:**
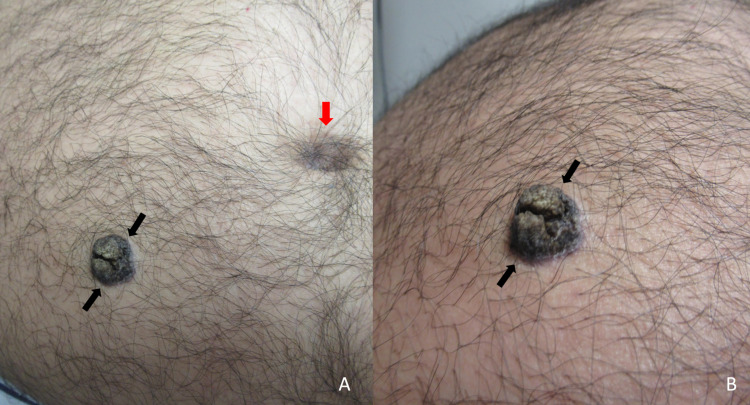
Clinical presentation of squamous cell carcinoma in situ mimicking a seborrheic keratosis. Distant (A) and closer (B) views of a single black plaque with fissures (black arrows) on the right lower abdomen, inferior to the umbilicus (red arrow), of a 62-year-old man.

A 62-year-old man presented for evaluation of a skin lesion that had been present for several years and changed in color and shape during the prior six months. The cutaneous examination was remarkable for a 1.5 x 2.0-centimeter hyperkeratotic black plaque, with fissures, on the right lower abdomen (Figure 1); a dermoscopic examination was not performed. There was no palpable axillary or inguinal adenopathy. The gross morphology of the tumor suggested a diagnosis of an inflamed seborrheic keratosis. A tangential shave biopsy attempting to remove the entire lesion was performed.

**Figure 2:**
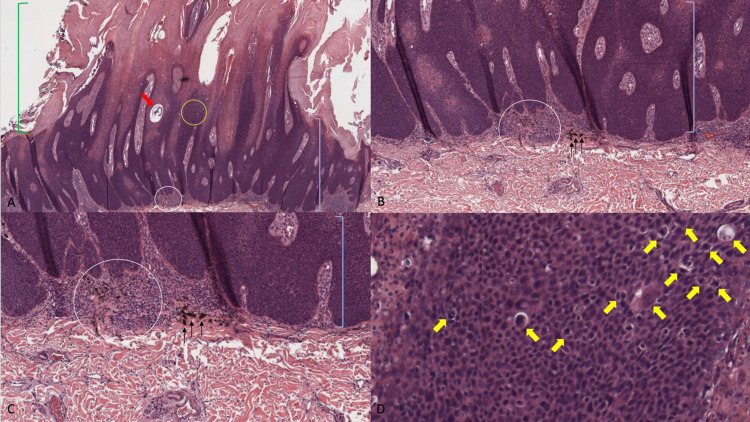
Microscopic examination of hematoxylin and eosin stained sections of squamous cell carcinoma in situ. Low (A) and higher (B and C) magnification views of the hematoxylin and eosin stained sections show hyperkeratosis (thickened stratum corneum; green brackets), acanthosis (thickened epidermis; blue brackets), and a pseudohorn cyst (red arrow). Lymphocytic inflammation (white circle) and melanophages (black arrows) are also noted in the papillary dermis. Higher magnification (D) of the epidermis (yellow circle in A) demonstrates several atypical keratinocytes (yellow arrows) (hematoxylin and eosin: A= x4; B= x10, C= x20, D= x40).

Microscopic examination of the biopsy tissue specimen showed thickening of the stratum corneum (hyperkeratosis), thickening of the epidermis (acanthosis), pseudohorn cyst in the epidermis, and hyperpigmentation. Lymphocytic inflammation and melanophages were present in the papillary dermis. At higher magnification, atypical nuclear features were present in keratinocytes throughout the epidermis (Figure 2).

Correlation of the clinical presentation and pathological findings established a diagnosis of a pigmented squamous cell carcinoma in situ. The residual tumor was excised. At the three-month follow-up, there has been no recurrence.

## Discussion

The pathogenesis of seborrheic keratosis has been postulated to grow in areas with exposure to ultraviolet radiation. Studies have shown that areas of skin with exposure to ultraviolet radiation have a higher expression of amyloid precursor protein in the epidermis than in non-exposed skin. The overexpression of amyloid precursor protein, as a result, may promote the growth of seborrheic keratoses [1]. 

Other theories of pathogenesis also include genetic and metabolic factors. Seborrheic keratosis has been described to be inherited in an autosomal dominant pattern with mutations of fibroblast growth factor receptor 3 (FGFR3) and the catalytic subunit p110 of phosphatidylinositol 3-kinase (PI3K). Metabolic causes may manifest as a paraneoplastic syndrome from gastric or colon adenocarcinomas, also known as the sign of Leser-Trélat, in which a sudden eruption of seborrheic keratoses can be observed [1]. 

Potential treatment modalities of seborrheic keratoses are varied, including observation. Most therapies are destructive in character; some of these include cryotherapy with liquid nitrogen, curettage (with or without desiccation), or conservative surgical removal with a scalpel or laser. Recently, a new treatment modality using topical application of hydrogen peroxide 40% to seborrheic keratoses has been introduced [1].

The incidence of seborrheic keratoses increases as patients become older. The appearance is variable with regards to color (such as flesh-colored, brown, or black) and morphology (such as a flat and macular patch-like lesion or a raised plaque with or without scaling) [1]. However, similar to the patient in this report, other tumors can clinically mimic the appearance of a seborrheic keratosis [1-19]. 

Biopsy-confirmed seborrheic keratoses can occasionally be misinterpreted clinically as skin cancers [20]; however, prior to biopsy, malignant neoplasms can mistakenly - based on morphologic features - be diagnosed as seborrheic keratoses [2,3,19]. In 1997, Eads et al. reviewed 577 pathology reports with the preoperative clinical diagnosis of seborrheic keratosis. Of the 577 suspected seborrheic keratoses based on morphologic features, 37 lesions (6.4%) were diagnosed as malignant tumors [2].

The following year, in 1998, Bryant published a similar study evaluating approximately 18,000 specimens (during a 30-month period from April 1, 1995 to September 30, 1997), of which 390 had a clinical diagnosis of seborrheic keratosis. Only eight of the 390 lesions (2.03%) were diagnosed histologically as malignant neoplasms. He concluded that the rate of malignant tumors misdiagnosed clinically as seborrheic keratoses that he observed was less than what Eads et al. had reported [19]. 

More recently, in 2017, Chen et al. analyzed 4361 cases clinically diagnosed as seborrheic keratoses or irritated seborrheic keratoses [3]. They found that 3759 (86.2%) were correctly diagnosed as seborrheic keratoses. However, 466 (10.7%) were determined to be non-malignant lesions and 136 (3.10%) were diagnosed as malignant tumors [3]. 

There are several cutaneous lesions that can masquerade as a seborrheic keratosis (Table 1) [1-18]. They include benign neoplasms and malignant tumors. They also include premalignant lesions. 

**Table 1:** Cutaneous lesions that can mimic seborrheic keratosis ^a^The autoimmune condition pemphigus foliaceus can also present with the new onset of multiple lesions that are hyperpigmented and appear like stuck-on verrucous papules and plaques that resemble eruptive seborrheic keratoses.

Lesion^a^	Neoplasm classification	References
Basal cell carcinoma	Malignant	[2,3]
Eccrine poroma	Benign	[4]
Epidermal nevus	Benign	[1]
Epidermolytic acanthoma	Benign	[5]
Hidroacanthoma simplex	Benign	[6]
Melanoacanthoma	Benign	[7]
Melanoma	Malignant	[2,3,6,8-11]
Mycosis fungoides	Malignant	[12]
Squamous cell carcinoma	Malignant	[2,3]
Squamous cell carcinoma in situ	Malignant	[2,3,13]
Superficial pigmented actinic keratosis	Pre-malignant	[14,15]
Verruca plana	Benign	[16]
Verruca vulgaris	Benign	[8]
Verruca xanthoma	Benign	[17,18]

Spreading pigmented actinic keratosis is a premalignant lesion that can also mimic seborrheic keratosis [14]. This dark-colored variant of actinic keratoses is typically found in sun-exposed areas and can spread laterally [15]. A 54-year-old man presented with an asymptomatic dark lesion on the nose tip that had been increasing in size over a one-year duration; a precancerous skin lesion was not suspected. However, microscopic examination of the punch biopsy demonstrated several atypical keratinocytes in the epidermis with overlying parakeratosis and immunohistochemical stains using melanoma antigen recognized by T-cells (MART-1) and microphthalmia-associated transcription factor (MiTF) did not show an atypical melanocytic proliferation. Thus, the diagnosis of pigmented actinic keratosis was established [15]. 

Benign tumors that mimic seborrheic keratosis can be adnexal (eccrine poroma and hidroacanthoma simplex), epithelial (epidermal nevus, epidermolytic acanthoma, and melanoacanthoma), viral (verruca plana and verruca vulgaris), or histiocytic (verruciform xanthoma). An eccrine poroma is a benign neoplasm that arises from the eccrine terminal duct. An 81-year-old woman with a verrucous nodule on her palm had the initial clinical and dermoscopic findings of seborrheic keratosis. However, histopathologic examination showed ductal structures, which revealed the lesion to be a pigmented eccrine poroma [4]. 

A 91-year-old woman with hidroacanthoma simplex on the flank that mimicked a seborrheic keratosis has been described. The lesion appeared similar to a seborrheic keratosis on physical examination and mimicked clonal type seborrheic keratosis on histology, but was ultimately differentiated to be an adnexal tumor by lumican staining. The immunohistochemical findings demonstrated diffuse lumican staining consistent with a hidroacanthoma, compared to sporadic lumican staining that would be found in a seborrheic keratosis [6]. 

Melanoacanthoma has been considered by some investigators to be a variant of seborrheic keratosis. Clinically, the lesion is morphologically similar to a thick keratotic seborrheic keratosis. However, a distinctive pathologic feature of this benign tumor is the presence of prominent dendritic melanocytes [7]. 

An 85-year-old man presented with several acquired skin lesions: a black nodule on his abdomen and black plaques on his left posterior shoulder and axilla. Microscopic evaluation of the tumor from the abdomen showed papillomatosis, acanthosis, pigmented melanocytes in the epidermis, and pigmented melanophages in the dermis, establishing the diagnosis of melanoacanthoma. In contrast, microscopic examination of the plaques from the shoulder and axilla demonstrated acanthosis with melanin deposits in the dermis, confirming the diagnosis of pigmented seborrheic keratoses. Therefore, he concurrently had not only a melanoacanthoma that mimicked a seborrheic keratosis, but also seborrheic keratoses [7]. 

An epidermal nevus, also referred to as an epidermal hamartoma, is an overgrowth of the epidermis. It can occur as an isolated lesion or as the cutaneous component of a syndrome that most often affects the brain, the eyes, and the skeletal systems. Not only the clinical morphology, but also the pathologic features of an epidermal nevus mimic those of a seborrheic keratosis; however, in contrast to a seborrheic keratosis, which is acquired later in life, an epidermal nevus is usually congenital and therefore present at birth [1].

Epidermolytic acanthoma can present as either a solitary lesion or multiple papules. Both clinical variants are morphologically similar to seborrheic keratosis. The pathology of epidermolytic acanthoma shows epidermal hyperkeratosis, which is a distinctive change in the epidermis that readily enables differentiation from seborrheic keratoses [5].

A 59-year-old woman presented with multiple hyperkeratotic vulvar papules. Her differential diagnoses included not only seborrheic keratoses, but also adnexal tumors, bowenoid papulosis, condyloma acuminata, and papular acantholytic dyskeratosis. However, the biopsy specimen showed papillomatosis with hyperkeratosis, hypergranulosis with clumped keratohyalin granules, and multifocal vacuolization of suprabasilar keratinocytes, which established the diagnosis of epidermolytic acanthoma [5].

Viral lesions that can mimic seborrheic keratoses include verruca plana and verruca vulgaris [8,16]. Verruca plana can be differentiated from seborrheic keratoses by their clustered distribution, Koebner’s isomorphic response, and red appearance within globular vessels on dermoscopy. An 80-year-old elderly woman presented with skin papules resembling seborrheic keratosis on her forehead. However, evaluation of the skin biopsy showed koilocytes and hypergranulosis in the upper epidermal layer, establishing the diagnosis of verruca plana [16]. 

A 48-year-old man had numerous warty and scaly exophytic lesions on the groin. Clinical and dermoscopic appearances were consistent with seborrheic keratoses. However, the histologic findings of one of the lesions established the diagnosis of verruca vulgaris [8].

Verruciform xanthoma is a benign histiocytic, papillary lesion that most commonly occurs on the oral mucosa; however, it can also occur in the genital region [17]. A 16-year-old adolescent girl presented with a pruritic verrucous lesion on the vulva of nine to 12 months duration. The clinical differential diagnosis included not only seborrheic keratosis, but also condyloma acuminatum, verruca simplex, verrucous carcinoma, and vulvar intraepithelial neoplasia. However, the pathologic findings of cells with foamy cytoplasm in the papillary dermis established the diagnosis of verruciform xanthoma [18]. 

Malignant tumors that can mimic a seborrheic keratosis include non-melanoma skin cancers, melanoma, and mycosis fungoides. Pigmented variants of non-melanoma skin cancer include basal cell carcinoma, squamous cell carcinoma in situ (Bowen’s disease), and squamous cell carcinoma. These cutaneous neoplasms are frequently misinterpreted as seborrheic keratoses [2,3,6,8-13,19]. 

Eads et al. reported that 37 specimens (6.4%) out of 577 clinically suspected seborrheic keratoses were actually biopsy-established malignant neoplasms. They included 19 squamous cell carcinomas, 12 basal cell carcinomas, four squamous cell carcinomas in situ, and two melanomas [2]. Chen et al. reviewed 4361 biopsy specimens with the clinical differential diagnosis of seborrheic keratosis or irritated seborrheic keratosis and found 136 (3.10%) malignancies; they included 91 in situ or invasive squamous cell carcinomas, 33 basal cell carcinomas, and 12 melanomas [3]. 

Another researcher, Bryant, studied 390 specimens with the clinical diagnosis of seborrheic keratosis. He discovered that only eight (2.03%) of the lesions that morphologically appeared to be seborrheic keratoses were actually non-melanoma skin cancers. He concluded that abstaining from biopsies of suspicious lesions may lead to delayed care of possible malignancies [19]. 

Sloan and Jaworsky conducted a study to evaluate the phenomenon of seborrheic keratoses transforming into malignant entities [13]. They reviewed 4310 specimens that were clinically diagnosed as seborrheic keratoses and found that 60 specimens (1.4%) were histologically proven to be squamous cell carcinoma in situ [13]. Although we do not consider our patient’s tumor to have undergone a malignant transformation from a seborrheic keratosis to a squamous cell carcinoma in situ, both clinical presentation and the low magnification appearance of the tissue specimen had several features consistent with an irritated seborrheic keratosis. 

There are several clinical and pathologic subtypes of melanoma. Pathologic changes of melanoma can rarely be simultaneously present with a seborrheic keratosis when both lesions concurrently exist as adjacent lesions (collision tumor) or mimic those of a seborrheic keratosis when the melanoma is seborrheic keratosis-like and has folliculotropism with dilated infundibula and keratin horny plugs in its epithelium. Verrucous melanoma refers to a seborrheic keratosis-like morphologic presentation of melanoma, which can be misinterpreted as a seborrheic keratosis [9,10]. 

Eads et al. reported two melanomas in the 527 specimens submitted as seborrheic keratosis [2]. Similarly, 12 melanomas were found from 4361 specimens reviewed by Chen et al. [3]. In a retrospective study by Izikson et al., 61 (0.66%) melanomas were discovered in their 9204 pathology reports with a differential diagnosis of seborrheic keratoses [11].

Albeit rare, a cutaneous lesion of mycosis fungoides that mimicked a seborrheic keratosis has been described. A 65-year-old man presented with an asymptomatic, slightly indurated pigmented, warty plaque on his left temple. Histology of the excisional biopsy specimen showed epidermal acanthosis with focal hyperkeratosis and parakeratosis, and a dense lymphoid infiltrate in the dermis with marked epidermotropism. Immunohistochemistry revealed CD3 positive T-cells and some CD30 positive cells, establishing the diagnosis of cutaneous T-cell lymphoma [12].

## Conclusions

Several benign, premalignant and malignant cutaneous lesions can clinically mimic a seborrheic keratosis. Therefore, if the patient presents with a morphologically changing seborrheic keratosis-like lesion, a biopsy may be considered to establish the diagnosis. Our patient’s skin lesion was clinically suspected to be an irritated seborrheic keratosis. However, a shave biopsy was performed, and microscopic evaluation of the biopsy specimen permitted the correct diagnosis of a squamous cell carcinoma in situ to be established. Similar to our patient, the pathologic features of tumors mimicking seborrheic keratoses permit them to be differentiated from seborrheic keratoses.
